# At the intersection of precision medicine and population health: an implementation-effectiveness study of family health history based systematic risk assessment in primary care

**DOI:** 10.1186/s12913-020-05868-1

**Published:** 2020-11-07

**Authors:** Lori A. Orlando, R. Ryanne Wu, Rachel A. Myers, Joan Neuner, Catherine McCarty, Irina V. Haller, Melissa Harry, Kimberly G. Fulda, David Dimmock, Teji Rakhra-Burris, Adam Buchanan, Geoffrey S. Ginsburg

**Affiliations:** 1 Department of Medicine, Center for Applied Genomics and Precision Medicine, Duke University School of Medicine, Durham, USA; 2 Programme in Health Services and Systems Research, Duke-NUS Medical School, Singapore, Singapore; 3 Department of Medicine, Medical College of Wisconsin, Milwaukee, USA; 4 Center for Patient Care and Outcomes Research, Medical College of Wisconsin, Milwaukee, USA; 5 University of Minnesota Medical School, Duluth campus, Duluth, USA; 6 Essentia Institute of Rural Health, Duluth, USA; 7 The North Texas Primary care Practice-Based Research Network and Family Medicine, University of North Texas Health Science Center, Fort Worth, USA; 8 Rady Children’s Institute for Genomic Medicine, San Diego, USA; 9 Genomic Medicine Institute, Geisinger, Geisinger, USA

**Keywords:** Risk assessment, Family health history, Genetic risk, Population health

## Abstract

**Background:**

Risk assessment is a precision medicine technique that can be used to enhance population health when applied to prevention. Several barriers limit the uptake of risk assessment in health care systems; and little is known about the potential impact that adoption of systematic risk assessment for screening and prevention in the primary care population might have. Here we present results of a first of its kind multi-institutional study of a precision medicine tool for systematic risk assessment.

**Methods:**

We undertook an implementation-effectiveness trial of systematic risk assessment of primary care patients in 19 primary care clinics at four geographically and culturally diverse healthcare systems. All adult English or Spanish speaking patients were invited to enter personal and family health history data into MeTree, a patient-facing family health history driven risk assessment program, for 27 medical conditions. Risk assessment recommendations followed evidence-based guidelines for identifying and managing those at increased disease risk.

**Results:**

One thousand eight hundred eighty-nine participants completed MeTree, entering information on *N* = 25,967 individuals. Mean relatives entered = 13.7 (SD 7.9), range 7–74. *N* = 1443 (76.4%) participants received increased risk recommendations: 597 (31.6%) for monogenic hereditary conditions, 508 (26.9%) for familial-level risk, and 1056 (56.1%) for risk of a common chronic disease. There were 6617 recommendations given across the 1443 participants. In multivariate analysis, only the total number of relatives entered was significantly associated with receiving a recommendation.

**Conclusions:**

A significant percentage of the general primary care population meet criteria for more intensive risk management. In particular 46% for monogenic hereditary and familial level disease risk. Adopting strategies to facilitate systematic risk assessment in primary care could have a significant impact on populations within the U.S. and even beyond.

**Trial registration:**

Clinicaltrials.gov number NCT01956773**,** registered 10/8/2013.

## Background

Population health is an increasingly important concept in U.S. healthcare systems. It has been around in various forms since civilizations began to understand the relationship between economic growth and human well-being [1]. It also serves as the basis for the preventive health guidelines that drive cancer and other disease screening recommendations in the U.S. For many decades, clinical medicine has operated essentially independently from population health, except for screening guidelines, prompting the Institute of Medicine to warn of the dangers of separation in their 1997 report [2]. With recent discussions about shifting healthcare payments from fee-for-service to value-based models, population health has become more of a priority topic in the U.S. However, our traditional approach to population health has been one-size fits all. For example, breast cancer screening was recommended for most women starting at age 40 until 2015 [3]. Unfortunately, several prominent examples, such as breast cancer screening, have shown that the broad application of a single rule across an entire population is fraught with harms – both in over- and under- screening and the attendant medical, psychosocial and financial costs.

Population management groups such as accountable care organizations use risk assessment to identify groups of individuals who need more intensive management (e.g., breast cancer surveillance at 30) and those who do not. Risk assessment separates a single large population into several sub-groups based upon their risk for developing any number of undesirable outcomes, such as a disease, hospitalization, or falls. Prevention and treatment strategies are then differentially applied to high and low risk groups. What is critical is that the process of risk assessment occurs at the level of an *individual* while the results lump individuals into *groups*. The acceptance of risk assessment as a valuable tool is demonstrated by its more frequent incorporation into recent guidelines. For example, use of cholesterol lowering medications for primary prevention of atherosclerotic cardiovascular disease (linked to 10-year risk≥7.5%) [4]; and adjunct use of breast MRI at age 30 for breast cancer surveillance in women with a lifetime risk of breast cancer > 20% [5].

Risk assessment at the individual level is a key element of precision medicine, which is broadly understood to focus on optimizing an individual’s health using genes, environment, and lifestyle to identify appropriate strategies for disease prevention, diagnosis, prognosis, and treatment [6]. Thus, risk assessment lives at the intersection of population health and precision medicine. Unfortunately, carrying out risk assessment is not well integrated into our current clinical care processes. The data needed to calculate risk, particularly family health history (FHH), an essential component that represents the most efficient way to clinically assess both genetic and environment impact on disease incidence is not routinely gathered, and when it is, providers are often unsure of what to do with it [7–10]. In addition, it is clear that the most effective place for risk assessment is the primary care setting; however, primary care providers are overwhelmed [11] and have little incentive or support to establish the infrastructure necessary to perform systematic risk assessment [12].

For these reasons there is a dearth of information about disease risk levels in the general U.S. population. Consequently, the health benefit that might be achieved if patients received preventive care matched to their level of risk is not realized. In this paper we describe the results of a study to systematically assess risk for 27 actionable conditions (a mix of common chronic diseases and hereditary syndromes) in primary care participants at four geographically and culturally diverse U.S. healthcare systems. This study is part of the Implementing Genomics in Practice (IGNITE) network (https://ignite-genomics.org/) funded by the National Human Genome Research Institute to facilitate the translation of genomic medicine into real world healthcare environments [13].

## Methods

### Intervention

Risk assessment in this study was performed using MeTree (https://goo.gl/uRSQWw), a patient-facing web-based risk assessment and clinical decision support program that interfaces with electronic medical records [14]. It collects personal and FHH data from participants for 98 medical conditions, as well as the personal data needed to run six validated calculators for breast cancer risk (BRCAPro [15], Gail Model [16], Tyrer-Cuzick Model [17]), and atherosclerotic cardiovascular disease (ASCVD) risk (Framingham ASCVD [18], Reynold’s risk score [19], and the pooled ASCVD risk equation recommended by the ACC/AHA guidelines [20]). Clinical decision support is automated, generating risk results and reports describing the evidence-based recommendations for any of the 18 different screening and prevention strategies the patient may meet criteria for (see measures and outcomes). Participants are provided extensive within-tool education on how and why to gather FHH information [21], as well as context sensitive help linked to Medline through the MedlinePlus Connect API. In addition, they are able to log in and out of MeTree as often as needed to address questions as they arise. The quality and quantity of FHH data collected has been previously reported and is considerably higher than what is typically available to the clinician when collected at the time of an appointment [22].

### Study design

This study was a pragmatic real-world implementation-effectiveness trial performed in 19 primary care clinics at four geographically and culturally diverse healthcare systems (University of North Texas Health Science Center (clinics comprised of largely migrant populations), Medical College of Wisconsin (clinics comprised of inner city and urban populations), Essentia Rural Health Institute (clinics located in very rural communities), Duke University Medical Center (clinics comprised of academic and suburban populations). Clinics in each healthcare system were enrolled using a delayed roll out process, and providers in the clinics opted into the study. Patients scheduled for routine appointments with participating providers were sent (via mail or email) a letter about the study 3 weeks prior of their appointment. Those who enrolled signed an electronic consent, completed an online baseline survey, and were given a web link to access MeTree. Risk results were provided to participants in real time as soon as data entry was completed, and to providers via the EMR. Study coordinators were available for assistance, if needed. Note that because this was a real-world implementation study, neither patients nor providers were offered any compensation for enrolling. The study was IRB approved by all institutions’ IRBs. See the published protocol for more details about the study [23], and the published implementation paper for study flow [24].

### Measures and outcomes

The baseline survey collected data on health literacy, medication adherence, lifestyle, participant’s health-related activation, readiness to change, and quality of life. For this paper health-related activation was measured using the validated patient activation measure (PAM) [25]. The PAM reflects a developmental model of activation with distinct stages of belief that the patient’s role is important; having the knowledge and confidence to take action; actually taking action; and the ability to stick with the health behavior change even when under stress. Scores range from 0 to 100 with 100 indicating high patient activation, which are classified into 5 levels with 5 being the highest level of activation.

MeTree collected the personal data needed to run the six risk calculators, as well as age, gender, race/ethnicity, twin status, consanguinity, diet, exercise, and medical conditions (and their age of onset). It collected FHH on live/dead status, if alive current age and if deceased cause and age of death, twin status, and medical conditions (and their age of onset). Participants were required to enter data on their parents and grandparents (6 relatives) but had the option of adding as many additional relatives as they wanted. We quantified both the number and types of relatives that were added as well as the amount of information entered for each.

MeTree generated the 5-year risk of breast cancer with the Gail Model, lifetime risk of breast cancer with Tyrer-Cuzick Model and BRCAPro, and 10-year cardiovascular risk. MeTree also indicated those who met guideline criteria for each risk management strategy (Table 2). These recommendations represent three levels of risk: 1) Monogenic hereditary conditions that have high disease penetrance associated with a single gene mutation (e.g. BRCA 1 mutation in Hereditary Breast and Ovarian Cancer Syndrome (HBOC)); 2) Familial risk that is considerably higher than the general population, but not as high as monogenic; 3) Common chronic disease risk, associated with the smallest increase in risk over the population level with odds rations of 2–3, is exemplified by diseases like atherosclerotic cardiovascular disease that have multiple environmental and genetic contributors with no single risk factor dominating.

### Analysis

Only participants who completed MeTree were included in this analysis. Demographics, family size, and number/type of relatives were summarized using counts and percentages or means and standard deviations. PAM quantitative scores were calculated and converted to an ordinal scale: 1 least activated to 4 most activated. Participant risk scores were summarized using means and standard deviations. Differences between healthcare systems were assessed using ANOVA F-tests.

FHH was summarized two ways: 1) proportion of families with at least one affected relative (*affected families*), and 2) proportion of relatives/family with the disease (*within families*), aggregated by participant, and summarized using means and standard deviations for family-wise proportions.

Clinical decision support results were aggregated by risk management recommendation (e.g., breast-MRI, genetic counselling referral, etc.) and summarized using counts and percentages. Additionally, total number of recommendations/participant were summarized using mean and standard deviation. Logistic regression modeled the presence/absence of a recommendation by patient demographics (race, ethnicity), healthcare institution, family size, and PAM score. Age and gender were excluded since these variables were part of the risk calculations and algorithms used to determine whether a patient met criteria for a recommendation, and, therefore, were not independent variables. Significance was assessed using likelihood ratio tests contrasting nested models, and generalized linear hypothesis testing was used to identify significant differences among pairs of categorical predictors with three or more levels.

## Results

Out of 2514 patients that consented and enrolled in the study, 1889 participants (75.1%) completed MeTree and received risk recommendations at the four healthcare institutions (Table 1**)**. Overall there were more women than men who enrolled in the study. There were no differences in gender by healthcare institution. In addition, most participants had some type of post-high school education and most were Caucasian. A manuscript on the study’s implementation outcomes describes the participants, providers and underlying clinic populations in detail [24].

**Table 1:** Participant Characteristics

Characteristic	N (%)
Gender	
Female	1310 (69%)
Male	578 (31%)
Race	
Caucasian	1607 (85%)
African American	143 (8%)
Asian	21 (1%)
American Indian/Alaskan Native	3 (0.2%)
Mixed Race	39 (2%)
Not reported	76 (4%)
Ethnicity	
Hispanic	35 (2%)
Non-Hispanic	1346 (71%)
Ashkenazi Jewish	76 (4%)
Not Reported	432 (23%)
Age in years	
< 50	537 (28%)
50–59	452 (24%)
60–65	293 (16%)
> 65	606 (32%)
Education	
High school or less	149 (8%)
Community college	276 (15%)
College	563 (30%)
Graduate or professional school	877 (47%)
Not reported	24 (1%)
Insurance	
Employer or private	1261 (68%)
Medicaid/Medicare	572 (30%)
Other	14 (1%)
Not reported	22 (1%)

### Data entered

**Fig. 1:**
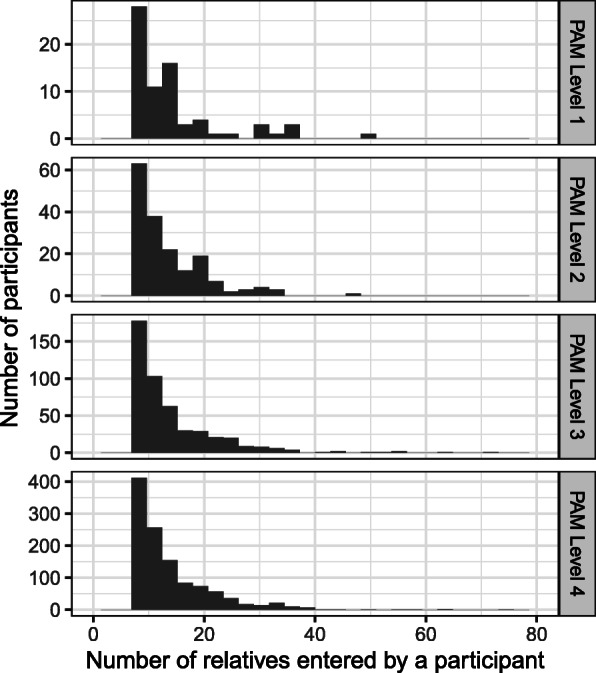
Distribution of the number of relatives entered by participants by their health care activation (PAM) level. Each panel represents the distribution of the total number of relatives entered by each participant with a PAM score at the designated level. The levels are shown on the bar on the left side of the image. The top panel represents participants who had the lowest PAM level, 1, and the bottom panel represents those with the highest PAM level, 4. The vertical axis on the right shows the number of participants and the horizontal axis the total number of relatives entered by each participant

The mean number of family members entered, including the participant, was 13.7 (SD 7.9) with a range of 7–74. Women entered significantly more relatives than men (mean 14.1 (SD 8.1) for women vs 12.9 (SD 7.1) for men). In total there were 25,967 relatives entered. Of these, 33.4% (*N* = 8688) were first degree relatives (FDR) (parents, siblings, and children), 53.2% (*N* = 13,822) second degree relatives (SDR) (aunts, uncles, grandparents, nieces, nephews, and grandchildren), and 6.0% (*N* = 1568) third degree relatives (TDR) (cousins). On a per-family basis, there was a mean of 4.6 (SD 2.3) FDRs, 7.3 (SD 4.7) SDRs, and 0.8 (SD 3.4) TDRs entered. Fifty percent of participants talked with relatives to collect FHH information. Additionally, they knew the medical history for 95% of FDRs, 70% of SDRs, and 54% of TDRs. Among relatives with a known medical history, a mean of 1.1 (range 0–10) diseases were entered for FDRs (though it was 2.6 (range 0–22) for the required FDRs- parents), 1.0 (range 0–10) for SDRS (1.5 (range 0–17) for required SDRs- grandparents), and 1.0 (range 0–8) for TDRs. In comparison, they entered 2.0 (range 0–15) for themselves. Whenever a disease was entered, 89.5% also had an age of onset entered. There was no difference in the number of relatives entered by PAM level (Fig. 1).

### FHH data

**Fig. 2:**
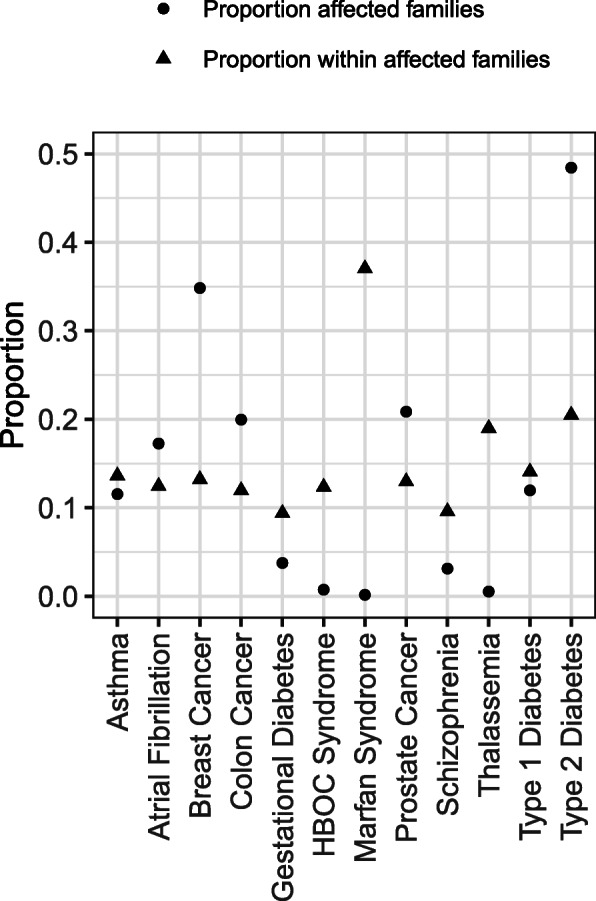
Proportion of families affected (affected families) by a condition and the proportion of family members affected (within family). The y-axis represents proportion with “Proportion affected families” represents the proportion of the 1889 families that contained at least one family member with the condition (as reported by the participants). “Proportion within family” represents the mean proportion of family members with the condition among families that have at least one affected family member

To describe the relationships of self-reported conditions in family units we selected several conditions for demonstration purposes. For these we report the proportion of families with at least one affected family member (*affected families*) and among affected families the proportion of family members affected (*within families*). These are shown in Fig. 2. The figure demonstrates the variation by disease and in particular highlights the clustering of some conditions within families. Higher proportions for “within family” indicate a higher degree of familial clustering (e.g. Marfan syndrome, thalassemia, hereditary breast and ovarian cancer (HBOC)). Figure 2 shows that, in some conditions (e.g. prostate and colon cancer), the proportion of affected families was higher than within families. In these cases the disease tended to be common and this characteristic outweighed the impact of familial relationships. Then there were some conditions for which *affected families* and *within families* were almost equivalent, such as asthma and type 1 diabetes. There were no differences between healthcare systems in the proportions for either *affected families* or *within families*.

### Health care activation survey results

The median and mean health-related patient activation measure (PAM) score was 70.8 (sd 13.9). When transformed *N* = 72 (4%) fell into level 1 (least activated), *N* = 174 (9%) level 2, *N* = 480 (26%) level 3, and *N* = 1154 (61%) level 4 (most activated). There were 9 participants who did not complete the survey. There were no differences between healthcare institutions.

### Risk results

Means and ranges for the 6 risk calculators were: 1) Gail 1.17% (0.18–6.08%). Missing data prevented calculations in 5/625 (5%), 2) Tyrer-Cuzick 8.16% (1.94–50.34%). Not calculated for 72/1022 (7%), 3) BRCAPro 8.08% (0.05–75.49%). Not calculated for 16/1507 (1%), 4) Framingham 8.7% (0–45%). Not calculated for 673/1507 (44%), 5) Reynold’s 2.89% (1.24–16.6%). Not calculated for 1447/1507 (96%), and 6) ACC/AHA 2.68% (0.02–32.35%). Not calculated for 694/1507 (36%). There were no differences in risk scores or missingness between healthcare institutions. Risk scores are shown in Table 2.

**Table 2:** Results of risk calculators

Risk Calculators	Mean Score (range)	# not calculated (%)
Gail Model for 5 year breast cancer risk	1.17% (0.18–6.08%)	5/ 625 (5%)
Tyrer-Cuzick Model for lifetime breast cancer risk	8.16% (1.95–50.34%)	72/ 1022 (7%)
BRCAPro	8.08% (0.05–75.49%)	16/ 1507 (1%)
Framingham 10 year CVD risk	8.7% (0–45%)	673/ 1507 (44%)
Reynold’s 10 year CVD risk	2.89% (1.24–16.6%)	1447/ 1507 (96%)
ACC/AHA 10 year CVD risk	2.68% (0.02–32.35%)	694/ 1507 (36%)

**Fig. 3:**
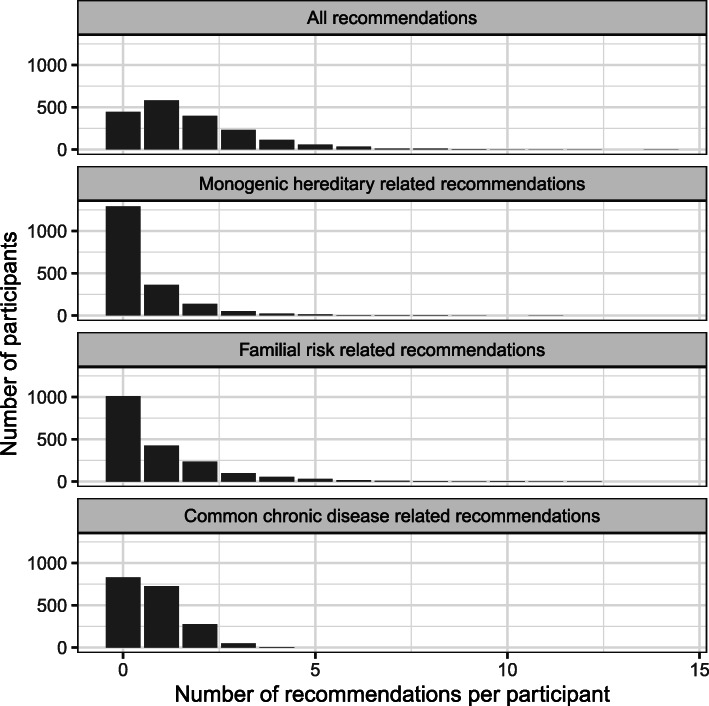
Distribution of the number of recommendations per participant for all recommendations, for only monogenic hereditary recommendations, and for common chronic diseases. The vertical axis represents the number of participants and the x-axis the number of recommendations per participant. Each panel shows the distribution of the number of recommendations per participant for “all recommendation types” (top panel), “monogenic hereditary syndromes” (second panel), “familial risk” (third panel), and common chronic diseases (bottom panel)

Table 3 shows the frequency with which individuals received a recommendation for a risk management strategy and were thus at increased disease risk for at least one condition. Overall 76.4% (*N* = 1443) of participants received at least one recommendation. 597 participants (31.6%) received a recommendation related to monogenic hereditary conditions, 508 (26.9%) for familial-level risk, and 1056 (56.1%) for risk of a common chronic disease. Among the hereditary cancer syndromes, the number of participants receiving recommendations for each type of syndrome was 246 for Hereditary Breast and Ovarian Cancer, 0 for PTEN Hamartoma Tumor Syndrome, 23 for familial adenomatous polyposis, and 71 for Lynch. In all there were 6617 recommendations given to the 1889 participants. The distribution of the number of recommendations received by each participant is shown in Fig. 3 for all recommendations, and broken down into only those related to monogenic hereditary conditions, familial risk, and common chronic disease risk. In a multivariate analysis of receipt of genetic counselling recommendations that adjusted for race, ethnicity, institution, total number of relatives entered, and activation score, there were no differences in risk recommendations except for total number of relatives entered (4.3% increase in the likelihood of receiving a risk recommendation for 1 additional relative entered, *p* < 0.001).

**Table 3:** Frequency of Risk Management Recommendations

Risk Management Recommendations	N (%)
**Monogenic Hereditary Related Recommendations**	**597 (31.6%)**
Genetic counseling for hereditary cardiac syndromes	107 (5.7%)
Genetic counseling for hereditary cancer syndromes [26–29]	395 (20.1%)
Genetic counseling for hereditary thrombophilia [30]	165 (8.7%
Familial hypercholesterolemia testing [31]	66 (3.4%)
Hemochromatosis iron studies and genetic testing [32]	3 (0.2%)
Wilson’s disease genetic testing [33]	11 (0.6%)
Alpha 1 anti-trypsinase deficiency genetic testing [34]	11 (0.6%)
**Familial Cancer Related Recommendations**	**508 (26.9%)**
Ovarian cancer screening discussion [35]	32 (1.7%)
Breast MRI screening [5]	58 (3.2%)
Breast cancer chemoprevention [36]	109 (6%)
Colonoscopy screening starting age < 50 and/or more frequently [37, 38]	241 (13.1%)
**Common Chronic Disease Related Recommendations**	**1059 (56.1%)**
Aspirin for stroke prevention [39]	67 (3.6%)
Diabetes screening	858 (45.4%)
Abdominal Aortic Aneurysm screening [40, 41]	389 (20.6%)
Calcium scoring CT for further cardiovascular risk stratification [4]	11 (0.6%)
Lung cancer screening [42]	45 (2.4%)

Because participants could receive more than one recommendation, percentages in the value column do not sum to 100

## Discussion

In this large multi-institutional study in diverse populations of a precision medicine tool for the systematic assessment of risk across 27 conditions, we found that a large percentage of the population (46%) is at hereditary or familial level of risk and meets criteria for more intensive risk management. This result might not seem extraordinary when considering common chronic diseases; however, it is significantly greater than what is widely perceived as the prevalence of hereditary (5%) [43] and familial risk (7–14%) [44] in primary care populations. Remarkably, despite the geographical and cultural differences in the participating healthcare systems (sites included rural, urban, and suburban environments; academic and private institutions, largely minority and largely Caucasian populations) (24), we found no differences in the percentage of participants at increased risk between the healthcare institutions, suggesting that these findings may translate across the broader U.S. population and potentially beyond as well given that risk assessment is not routinely collected in many healthcare systems across the globe [45, 46].

Our focus in this study was the implementation of systematic risk assessment. To systematically assess risk there are several requirements. First, patients should be unselected; everyone should undergo risk assessment. Second, a high quality personal and FHH is essential for accurate risk assessment as many guidelines, such as the National Comprehensive Cancer Network’s HBOC risk assessment guidelines, are based entirely on personal FHH [26]. The five elements needed to meet a high quality designation are: 1) relative’s gender and relationship to the patient (i.e. aunt, male cousin), 2) relative’s side of the family (maternal/paternal), 3) alive/dead status, 3) age, if living, and age and cause of death, if not, 4) relative’s medical conditions (all) with the age of onset for each, and 5) at least three generations of relatives (at a minimum parents and grandparents) [47]. Third, the process should be initiated automatically without the need for manual curation. Optimally this occurs electronically through a patient portal; but could also be part of a pre-appointment intake on a waiting room computer or tablet, if patients come prepared with their family’s health history collected ahead of time. Lastly, data need to be analyzed into clear guideline-based risk management strategies available in the medical record for the provider or clinic support staff at the time of care. Currently, even the most advanced EMRs only partially support one of these requirements- data collection through the patient portal. In this instance, providers can manually send a FHH questionnaire to the patient prior to their appointment.

In this study, participants entered a considerable amount of medical data on a large number of relatives. We have seen this in our prior studies as well. When participants are educated about why and how to collect FHH, and given the time to collect it, the quantity and quality of FHH data provided exceeds that which can be gathered at the point of care during a primary care visit [21, 22, 47]. Accuracy of FHH collected using patient-facing risk assessment platforms is frequently comparable to that collected by genetic counsellors, which is commonly considered the gold standard [48–50]. Overall, most participants entered considerably more information than what was required by the software. For example, only parents and grandparents were required but the mean number of relatives entered was 13.7 with some entering up to 74. Most also gathered the data needed to run the risk scores. We were able to run the breast cancer risk scores on almost all participants eligible for the score (93–99% depending on the score), and most gathered and entered lab values for the cardiovascular risk calculators (64–66%). The low calculation rate for the Reynold’s risk score (3%) was primarily due to not having a c-reactive protein result. This is unsurprising since it is not a routinely ordered test.

Interestingly, the total number of relatives input was correlated with identifying an increased risk for a condition. This warrants further investigation. Exactly how many and what type of relatives are needed to optimize risk assessment? Did participants with more disease in their families enter more relatives because they were primed by their “bad” FHHs, or is there important information that could not be otherwise gleaned except by adding more relatives? We attempted to evaluate this question by looking at the relationship between healthcare activation (PAM level) and the number of relatives participants entered, theorizing that participants with worse FHHs might be motivated to enter more data and may also have higher PAM scores. Overall, participants had high PAM scores, a finding we attribute to those willing to enroll in risk-based studies since feeling in control of your own health is an important step in managing risk; however, we did not see a significant difference between the number of relatives entered when stratified by PAM level (Fig. 1). In addition, it was not associated with receiving a high risk recommendation.

Another interesting finding in this study was the correlation between proportion of affected families and clustering of conditions within families. This difference is demonstrated in Fig. 2. High prevalence within affected families is seen, as would be expected, in conditions with known monogenic hereditary inheritance patterns, such as Marfan’s, thalassemia, and HBOC; however, these conditions are considered rare and the family clustering is evidenced by the very high within family proportion as compared to the low proportion of affected families. Surprisingly there were also conditions where the proportion of affected families was very high but there was minimal clustering within families, such as type 2 diabetes mellitus. Since FHH represents the impact of both shared genetics and shared environment, identifying whether these relationships are due to genetics alone, environment alone, or the combination of genetics and environment is worth exploring in more detail. This is yet one more reason that collecting FHH should be a priority in health care.

As with all studies, this study has limitations. An important question is whether the study population is truly representative of the general primary care population or if it may be biased. For example, were individuals with strong family histories of disease more likely to enroll in a risk assessment study than those without? It is hard to fully answer this question as we do not have access to FHH data, education, income, or other important variables on the underlying clinic population. A detailed analysis of the intervention’s implementation and the implementation outcomes, including participant uptake as compared to the general clinic population, is published and shows that there are some differences, such as participants were more frequently females, older, and Caucasian than the underlying clinic population [51]. For minorities who did enroll in the study, we found they were equally likely to complete the study (with the exception of Asians) and the quality of their data was equal to that of the overall study population [43]. This is supported by prior studies as well which indicate minorities have equal access to mobile devices and are equally likely to use mHealth applications [52, 53]. The challenge of how to engage these patients in the beginning remains to be addressed, but it is reassuring that once engaged there do not appear to be racial barriers to completion of risk assessment. These are often limitations of research studies, yet one effective way to address this barrier, since risk assessment is recommended as part of clinical care, would be to incorporate systematic risk assessment into clinical care and monitor outcomes as part of a quality improvement initiative. Another limitation of this study is the reliance upon self-reported personal and FHH information. We were not able to obtain relatives’ medical records to verify medical data; however, previous studies have shown that self-report using FHH software programs is significantly better than FHH collection methods currently available in clinical practice, and in some cases has even been shown to be equivalent to what is collected by genetic counselors [54]. In addition, the frequency with which lab data for the cardiovascular risk calculators was collected and entered suggests that patients can be highly motivated to gather appropriate data.

One additional caveat to consider is that not all providers will agree with the guidelines applied in the study population. This is not unexpected and the autonomy of providers to decide which guidelines have reached the level of evidence to garner their support is an important part of medical practice. The hereditary-based guidelines have relatively broad acceptance with no competing guidelines available at this time, though, the familial and common chronic disease guidelines are widely debated. Our purpose in the study was not to say that all providers should follow the specific guidelines assessed; but to instead highlight the need for a systematic process for assessing risk. Regardless of the guidelines we choose to follow our patient populations are not one size fits-all. They are remarkably diverse with most individuals having increased risk for something- measuring that risk and tailoring prevention strategies is an essential step for moving our healthcare systems forward.

Prior to this study, none of the participating healthcare systems had a method for systematic collection of FHH or risk assessment in primary care. In fact we know of no institution that does. In light of the high numbers of participants from the general population in our study who met increased risk management criteria for hereditary and familial conditions, we believe there is considerable opportunity to improve health outcomes in the U.S. population if institutions develop strategies to address this gap.

## Conclusions

A significant percentage of the general primary care population as observed in this study and similar studies meet criteria for more intensive risk management. Overall, 46% meet criteria for monogenic hereditary and familial level disease risk with a substantial percentage, 26.9% in the hereditary category. The limitations of collecting and analyzing family history in primary care settings hinders early identification of these individuals and initiation of guideline-based preventive/risk reductive care. Adopting strategies to facilitate systematic risk assessment in primary care could address these limitations and have a significant impact on population health.

## Data Availability

The data collected during this study and the analyses used in this study are reported in this paper. All de-identified data from the study has been deposited in the dbGaP public database as required by NIH.

## Abbreviations

FHH: Family health history
IGNITE: Implementing Genomics in Practice network
API: Application programming interface
EMR: Electronic medical record
IRB: Institutional review board
PAM: Patient activation measure
FDR: First degree relative
SDR: Second degree relative
TDR: Third degree relative
HBOC: Hereditary breast and ovarian cancer
